# Thrombosis and Anticoagulant Therapy Among Pediatric Cancer Patients: Real-Life Data

**DOI:** 10.7759/cureus.20084

**Published:** 2021-12-01

**Authors:** Hasan Hashem, Momen Zeineddin, Rayan Bater, Nisreen Amayiri, Wiam Al Qasem, Bilasan Hammo, Iyad Sultan, Rama AlMasri, Hikmat Abdel-Razeq

**Affiliations:** 1 Department of Pediatrics, King Hussein Cancer Center, Amman, JOR; 2 Department of Internal Medicine, King Hussein Cancer Center, Amman, JOR; 3 Department of Pharmacy, King Hussein Cancer Center, Amman, JOR; 4 Department of Internal Medicine, Istishari Hospital, Amman, JOR; 5 Department of Medicine, School of Medicine, University of Jordan, Amman, JOR

**Keywords:** thromboprophylaxis, anticoagulation, venous thromboembolism, pediatrics thrombosis, cancer

## Abstract

Background: Venous thromboembolism (VTE) in children is relatively rare, and more so among those with cancer. In this study, we report the characteristics and outcomes of children with cancer-associated thrombosis.

Methods: We reviewed institutional databases for all children with cancer and a diagnosis of VTE at King Hussein Cancer Center in Jordan. Variables reviewed are patients’ clinical characteristics, treatment for cancer, and anticoagulation therapy.

Results: Between January 2011 and December 2018, a total of 45 patients fulfilled the inclusion criteria, and the median age was 10.4 (0.8-17.9) years. The most common underlying diagnosis was acute lymphoblastic leukemia (n = 13, 29%). At the time of VTE, 29 (64.4%) patients were receiving chemotherapy, and eight (17.8%) had a central venous catheter (CVC). The majority of patients (n = 37, 82%) developed VTE within 30 days of hospitalization. Thrombosis mostly involved the extremities (n = 23, 51%) and sagittal vein (n = 12, 26.7%). All patients were treated with low-molecular-weight heparin (LMWH), complicated by bleeding in three (6.6%) patients.

Conclusion: In contrast to adults, VTE in pediatric cancer patients is more associated with chemotherapy and recent hospitalization. LMWH is a safe and effective therapy for children with cancer who develop VTE.

## Introduction

Venous thromboembolism (VTE), including deep vein thrombosis (DVT) or pulmonary embolism (PE), is a known complication of malignancy [1]. The majority of studies investigating incidence, risk factors, and treatment of VTE in the setting of cancer have focused on the adult population [2,3]. While VTE is rare in children with cancer, it has been increasingly diagnosed [4-8].

Children with cancer are at increased risk of VTE compared with the general pediatric population. The incidence of VTE in children with cancer ranges from 2.0 to 16.0% [9,10,11]. VTE in children with cancer is multifactorial and includes disease-related factors (type of cancer, inflammation, and abnormal blood flow) and treatment-related factors (presence of central venous catheters [CVC], surgery, and type of chemotherapy) [12,13].

The majority of data on the treatment of VTE in children with cancer has been extrapolated from adults literature and observational studies in the general pediatric population. Nevertheless, there have been recent guidelines from the Children’s Oncology Group and the American Society of Hematology specific to children with cancer, and low-molecular-weight heparin (LMWH) is the anticoagulant of choice [14]. The significant burden of VTE complications, including potential worsened prognosis, seems to justify that certain patients with cancer and high-risk features may be considered for primary thromboprophylaxis. However, to date, there have been no clear guidelines in pediatric cancer patients with regard to thromboprophylaxis. In addition, many pediatric oncologists are hesitant to use prophylactic anticoagulation in children with cancer because of the associated risk of bleeding, mostly due to pre-existing thrombocytopenia [15].

The majority of information on VTE in children with cancer originates from studies conducted in children with acute lymphoblastic leukemia (ALL) [9,16]. Other studies evaluated VTE in pediatric patients with various oncologic diagnoses [10,17-19]. Hence, we conducted a retrospective study to evaluate the characteristics and treatment of VTE among pediatric patients with cancer at King Hussein Cancer Center, Amman, Jordan.

This article was previously presented as a poster at the 2020 ISTH Virtual Congress on July 11, 2020.

## Materials and methods

This is a retrospective single-center study. Medical records and electronic institutional and pharmacy databases were reviewed for all children < 18 years of age, with a diagnosis of cancer, who received any form of anticoagulation. We included pediatric patients with cancer and VTE. All VTE events were radiologically confirmed. Variables collected include patients’ age, gender, primary cancer type, type of anticancer therapies (surgery, chemotherapy, and radiotherapy) in addition to type and duration of anticoagulation used, and associated complications. Descriptive statistics were performed for all variables. Continuous variables with normal distribution were presented as mean (standard deviation [SD]); non-normal variables were described as median (interquartile range [IQR]), and categorical variables were expressed as numbers (percentages). Results for continuous variables are expressed as median (range), and categorical variables are expressed as numbers (percentage). This study was approved by the Institutional Review Board (IRB) at King Hussein Cancer Center (KHCC) (approval number: 18-KHCC-107).

## Results

From January 2011 to December 2018, 45 eligible pediatric patients with cancer and radiologically confirmed VTE were identified and included in our study. All were younger than 18 years at the time of cancer diagnosis. The median age at VTE diagnosis was 10.4 (range 0.8-17.9) years, and 22 (48.9%) were males. The patients’ characteristics are detailed in Table 1.

**Table 1:** Patients characteristics (n = 45) RMS, Rhabdomyosarcoma.

Characteristics	Number	Percentage
Age (years)		
Median (range)	10.4 (0.8-17.9)	
Primary tumor		
Leukemia	13	28.9
Lymphoma	6	13.3
Bone	6	13.3
Brain	6	13.3
RMS	3	6.7
Others	11	24.5
Chemotherapy	40	88.9
Radiotherapy	3	6.7
Anticoagulation prophylaxis	0	0
Surgery	7	15.5

Risk factors for VTE

The median time from diagnosis of cancer to VTE was 30 (1-3333) days. Most (n = 37, 82.2%) patients were hospitalized within 30 days of VTE occurrence. Eight (17.7%) patients had CVC, seven (15.5%) had major surgery, and three (6.7%) had radiation therapy before VTE diagnosis. Twenty-nine (64.4%) patients had received chemotherapy within one month of VTE; 23 (79.3%) of them developed VTE during the first month of chemotherapy.

Site of VTE

The most common site for VTE formation was the lower limbs (n = 21, 46.6%), followed by cerebral sagittal sinus venous thrombosis (CSVT) (n = 12, 26.6%). Two patients developed upper extremity DVT. PE was diagnosed in four patients, two of which had lower extremity VTE (Table 2).

**Table 2:** Venous thromboembolism (VTE) characteristics *Two patients developed both lower extremity DVT and PE. CSVT, Cerebral sagittal sinus venous thrombosis; PE, pulmonary embolism; DVT, deep vein thrombosis.

Clinical Variables	Number of Patients	Percentage
VTE within 30 days of last chemotherapy	29	64.4
VTE within 30 days of admission	37	82.2
VTE Site		
Lower extremity	21*	46.7
Upper extremity	2	4.4
PE	4*	8.8
CSVT	12	26.7
Others	8	17.8
Complications		
Minor bleeding	1	2.2
Major bleeding	2	4.4
Thrombocytopenia	14	31.1
None	28	62.3

CSVT was diagnosed at a median age of 11.7 (2.2-16.7) years. Of the 12 patients with CSVT, five (41.6%) had B-ALL, three (25.0%) had a brain tumor, while Hodgkin lymphoma and synovial sarcoma were diagnosed in two patients (Table 3). Nine (75.0%) of 12 patients were on steroids at the time of VTE diagnosis. All B-ALL patients received steroids and asparaginase and were either on induction or consolidation chemotherapy.

**Table 3:** Clinical characteristics of patients with CSVT (n = 12) CSVT, Cerebral sagittal sinus venous thrombosis.

Characteristics	Number	Percentage
Age (years)		
Median (range)	11.7 (2.2-16.7)	
Primary tumor		
Leukemia	5	41.6
Brain	3	25
Hodgkin lymphoma	2	16.7
Synovial sarcoma	2	16.7
Chemotherapy	11	91.7
Radiotherapy	1	8.3
Anticoagulation prophylaxis	0	0
Surgery	3	25.0

Treatment of VTE

All VTEs were treated with Enoxaparin at a dose of 1 mg/kg/dose twice daily, and none were on thromboprophylaxis before the diagnosis of VTE. Anti-Xa measurements are not available at our institution, and hence, all patients remained on the same dose of Enoxaparin throughout the treatment duration. Duration of anticoagulation varied from three months in 21 (46.6%) patients, six months in nine (20%) patients, and 12 months in 13 (28.8%) patients (Table 2). Three patients (6.7%) developed bleeding as a complication of anticoagulation; two were major, and all have recovered. Recurrent VTE was encountered in two patients, and post-thrombotic syndrome was reported in one patient.

## Discussion

In this retrospective study, we highlight a real-life experience addressing the issues related to diagnoses, treatment, and associated complications encountered in pediatric cancer patients over an eight-year period.

Leukemia was the most common cancer among pediatric patients who developed VTE, in agreement with other reports [18]. Although leukemia is the most common cancer associated with VTE, the majority of our cohort (70%) had other types of cancer. Unlike adults with brain tumors, VTE in children with brain tumors is quite low; we observed only (1.7%) consistent with other reports (0.64%-2.8%) [20-22].

A great deal of debate remains on the risk factors associated with VTE in pediatric cancer patients [18,23,24]. In the present study, we have identified asparaginase use concomitantly with steroids as a risk factor for VTE in B-ALL patients in-line with the published literature [9,16,25,26]. Although our study did not specifically look into all the risk factors associated with VTE due to its retrospective nature, larger prospective studies incorporating all potential risk factors associated with VTE are needed to solve the query.

The current study confirms previous observations that VTE is most frequently observed during chemotherapy [23]. Almost half of VTE events occurred during the first month of therapy, and two-thirds of events occurred within two months. This observation, if confirmed, potentially has important implications in designing screening and interventional strategies in terms of thromboprophylaxis for pediatric cancer patients.

Another important observation is that LMWH was effective in managing VTE in pediatric cancer patients. As all our patients with VTE were treated with Enoxaparin, only two patients (4.4%) developed recurrent VTE events. The rate of recurrence in our study is lower than that reported in the literature (13.8%) [19]. Anticoagulation was complicated by bleeding in only three patients (6.6%), one minor and two major bleeding; all have recovered. LMWH was safe and effective for the management of VTE in pediatric cancer patients.

The data on thromboprophylaxis in children with cancer is scarce [15,27]. Specifically, in ALL, three studies have evaluated the use of LMWH at the start of asparaginase treatment and until one week after the last dose. In a prospective cohort study of 41 children with ALL, Elhasid et al. showed that LMWH might be effective [28]. Meister et al. compared the effect of anti-thrombin (AT) substitution alone versus LMWH; VTE occurred in nine (12.7%) of 71 patients with AT substitution alone, while no VTE occurred in 41 patients with AT plus LMWH (p = 0.02) [29].

The authors concluded that LMWH prophylaxis was safe and effective. Harlev et al. administered LMWH only to patients with inherited thrombophilia and ALL and concluded that routine evaluation of thrombophilia followed by thromboprophylaxis to those who screen positive may benefit the at-risk patients with ALL [30]. Although thromboprophylaxis seems to be safe, the available literature is not convincing its routine use in children with cancer. However, it may be reasonable to use thromboprophylaxis in certain high-risk situations with a careful balance between risk and benefit. The Children’s Oncology Group anticoagulant prophylaxis phase 3 study (ACCL 1333) is currently testing the efficacy of Apixaban for primary VTE prevention versus no anticoagulation during induction chemotherapy in children with newly diagnosed ALL treated with asparaginase [25].

Although our study has considerable limitations in terms of the retrospective design, heterogeneous group of patients and diagnoses, and the inclusion of symptomatic VTE events only, our study catches all patients with symptomatic VTE in our institution. Moreover, our study did not specifically assess all risk factors associated with VTE due to its retrospective nature and limited sample size. Our data suggest that, unlike adults where most VTE events occur in the ambulatory setting, most VTE events relate more to recent hospitalizations and chemotherapy administration. Moreover, our data show that usage of steroids concurrently with asparaginase might increase the risk of VTE, more so for leukemia patients. LMWH is safe and effective in VTE management in pediatric cancer patients. These findings set the stage for future evaluations with prospective studies to evaluate the role of thromboprophylaxis for leukemia patients receiving concurrent steroid and asparaginase during induction and consolidation chemotherapy.

## Conclusions

Compared to adults, thrombosis in pediatric patients, even among those with cancer, is relatively rare. Recent hospitalization and treatment with chemotherapy are major risk factors. In addition, using steroids concurrently with asparaginase might increase the risk of VTE. Anticoagulation with LMWH is effective and safe in the treatment of thrombosis in pediatric oncology patients. Larger prospective studies incorporating all potential risk factors associated with VTE are needed to solve the query. Although thromboprophylaxis seems to be safe, the available literature is not convincing its routine use in children with cancer, and it may be reasonable to use thromboprophylaxis in certain high-risk situations with careful balance between risk and benefit.
